# Self-Report Dieters: Who Are They?

**DOI:** 10.3390/nu11081789

**Published:** 2019-08-02

**Authors:** Laura Sares-Jäske, Paul Knekt, Satu Männistö, Olavi Lindfors, Markku Heliövaara

**Affiliations:** 1 Department of Public Health Solutions, National Institute for Health and Welfare, 00271 Helsinki, Finland; 2 Department of Public Health, University of Helsinki, 00014 Helsinki, Finland

**Keywords:** dieting attempts, intentional weight loss, determinants, obesity

## Abstract

Dieting attempts have become popular worldwide. Dieting, however, seems to have both positive and negative health-related consequences. So far, only a few studies have focused on the determinants of dieting in detail. This study explores the association between self-report dieting attempts and intentional weight loss (IWL) during the previous year and several demographic, lifestyle, health, and psychological factors in a cross-sectional study design using data from the representative Finnish Health 2000 Survey. The sample comprised 2147 men and 2378 women, aged 30–69. Information for potential determinants was assembled via health examinations, interviews, and questionnaires. Approximately 24% of the men and 39% of the women reported dieting attempts and 10% of the men and 15% of the women reported IWL. Dieting attempts were associated with younger age, education, BMI, formerly smoking, more favourable values in lifestyle variables, and unfavorable values in serum HDL and triglycerides, a worse sense of coherence, concerns about one’s appearance, and concerns about one’s health. Among men, diabetics and those sleeping ≤6 h a night more frequently reported dieting attempts and those with osteoarthritis reported IWL. Moreover, the gradient between BMI and dieting attempts was significantly stronger in men than in women. Men seem to attempt dieting when they have actual health-related reasons, while such reasons are not so strongly associated with dieting in women. These findings can be used for determining subpopulations with obesity and real weight-loss needs and, alternatively, subpopulations with normal weight unnecessarily attempting dieting.

## 1. Introduction

Obesity acts as a major burden on public health by increasing the risk of several chronic diseases, such as cardiovascular diseases, type 2 diabetes (T2D), some cancers, osteoarthritis, and depression [1]. Accordingly, dieting is used as a prevention strategy against the occurrence of such diseases and, indeed, successful and sustained weight loss benefits individuals with obesity [2,3]. Longitudinal epidemiological studies, however, have shown subsequent excess weight gain among dieters [4,5,6,7]. Moreover, dieting attempts may have other adverse consequences by inducing weight cycling, which has been suggested to be related to fluctuations in metabolic and cardiovascular risk factors (e.g., blood pressure, serum lipids, and plasma glucose) and the elevated risk of metabolic syndrome [8,9]. In general, the known health consequences of weight cycling seem to remain inconsistent [10,11].

In addition to health-related reasons, individuals attempt dieting due to appearance, sport activities, and social or cultural pressure. Overall, more than 40% of the adult population worldwide have reported dieting attempts at some point in their life [12]. In spite of such popularity, however, studies on dieting attempts and intentional weight loss (IWL) at the population level have been relatively scarce.

In the light of the high prevalence and possible contradictory consequences, it is essential to study the distribution of dieting according to relevant determinants in order to be able to identify the persons who report dieting. This knowledge could be utilized in preventing possible weight gain potentially resulting from unnecessary dieting. Moreover, information on the determinants of dieting is needed to be able to assess, without bias, whether dieting predicts the occurrence of non-communicable diseases. Previous studies have shown that the prevalence of dieting attempts or intentional weight loss (IWL) varies with sex [12,13], age [14,15,16,17,18], education [14,15,16,17,19,20], income [14,20,21], physical activity [22,23,24,25], weight [12,13], and indicators of dietary habits [24,26,27,28,29,30,31]. However, there is a need for studies that simultaneously cover socio-demographic factors, lifestyle, metabolic biomarkers, somatic diseases, and mental health factors at population level.

This is the first study that simultaneously explores a comprehensive set of factors for their associations with dieting in a nationally representative general adult population.

## 2. Materials and Methods

### 2.1. Study Population

The present study is based on the Health 2000 Survey (BRIF8901) carried out during 2000–2001 [32]. The sample, representative of the Finnish adult population, was drawn with a two-stage stratified cluster sampling design from 80 districts in mainland Finland. The sample included 8028 individuals aged 30 years and over. Of this sample, 6771 (84% of the sample) took part in a health examination. The sample used in this study comprised 4525 individuals (2147 men and 2378 women) who were 30–69 years old, not pregnant, had measured BMI information available, and had information available on dieting attempts and weight loss during the previous year.

### 2.2. Methods

#### 2.2.1. Dieting Variables

Information on dieting attempts (‘Have you tried to lose weight during the last 12 months? No/Yes’) and weight loss (‘Have you lost weight during the last 12 months? No/Yes’) during the previous year was assessed using a self-administered questionnaire. An IWL variable was created by combining the two variables; participants answering ‘yes’ to both questions were recoded as having IWL. Although an additional question concerned the amount of lost weight, we included all individuals who had tried to lose weight and had lost weight as those with IWL, regardless of the amount of weight lost during the previous year (any amount from 1 kg upwards).

Data on the potential determinants of dieting was drawn from questionnaires, interviews, a health examination, and national registers.

#### 2.2.2. Socio-Demographic Factors

Information concerning sex, age, and residential area was collected from national registers. A residential area was divided into urban town, densely populated municipality, and rural municipality. Information on education and marital status was collected with an interview. Education was categorized as a three-class variable, as follows: Low (did not graduate from upper secondary school or vocational school), intermediate (graduated from upper secondary school or vocational school), and high (graduated from university or vocational college). Marital status was divided into four categories, as follows: Married or cohabiting, divorced or separated, widowed, and single.

#### 2.2.3. Lifestyle-Related Factors

Data for anthropometric measurements was measured at the health examination while wearing light clothing and no shoes. Height (cm) was measured with a wall-mounted stadiometer with the participant standing and with a precision rate of 0.5 cm. Weight (kg) and fat free mass (kg) were measured with an eight-polar bioimpedance device (InBody 3.0, Biospace, Seoul, South Korea). The results were recorded with an accuracy of 0.1 kg. The BMI was calculated as weight (kg) divided by the square of the height (m^2^). Normal weight was defined as BMI < 25 kg/m^2^, overweight as BMI 25–29.9 kg/m^2^, and obesity as BMI ≥ 30 kg/m^2^ [33]. As the sample included only 29 individuals who were underweight (BMI < 18.5 kg/m^2^), they were combined with those who had normal weight. A fat free mass index (FFMI) was calculated as fat free mass (kg) divided by the square of the height (m^2^). Leisure-time physical activity was measured via a self-administered questionnaire and divided into three categories, as follows: Low physical activity (those not physically active), moderate physical activity (those regularly engaging in light physical activity like walking or cycling), and regular physical training (those reporting exercising for three hours or more per week or training for competitive sports).

Data on sitting time was derived from a self-administered questionnaire. The participants were asked how many hours they sit during an ordinary weekday and weekend day. Sitting time on a weekday was multiplied by five and sitting time on a weekend day was multiplied by two. The products were summed together and divided by seven. The average daily sitting time was further divided into sex-specific tertiles.

Information on smoking was collected by interviews [32]. Individuals were categorized into never smokers, former smokers, and current smokers.

The habitual diet was measured with a self-administered food frequency questionnaire (FFQ) assessing food intake over the last 12 months [34,35]. The National Food Composition Database (Fineli^®^) and in-house software (Finessi) [36] were used to calculate the average daily intake of food groups, energy, and nutrients. The Alternate Healthy Eating Index (AHEI) [37] was used as a measure of the quality of the diet. The AHEI used in this study was composed to imitate the original AHEI as closely as possible, while paying attention to the special characteristics of the Finnish dietary culture [38]. Information on the daily consumption of certain sugary products was collected through a questionnaire. The questions concerned the consumption of (1) juices, soft drinks, and hot chocolate, (2) toffee, licorice, and dried fruit (e.g., raisins), (3) sweets, hard pastilles, and candy without xylitol, and (4) chocolate and filled biscuits. Each question included the response options ‘3 times a day or more often’, ‘Once or twice a day’, ‘2 to 5 times a week’, ‘More rarely’, and ‘Never’. The alternatives ‘3 times a day or more often’ and ‘Once or twice a day’ for any of the products were coded as daily consumption.

Information about the average sleep duration during 24 h was asked on a questionnaire. Sleep duration was categorized as ‘≤6 h’, ‘7–8 h’, and ‘≥9 h’.

Alcohol consumption (grams ethanol/week) was measured on a questionnaire and was divided into non-users, moderate users (1–199 g ethanol/week for males and 1–99 g ethanol/week for females) and heavy users (200 g ethanol/week or over for males and 100 g ethanol/week or over for females).

#### 2.2.4. Somatic Health

Serum triglycerides (automated enzymatic method, Olympus system reagent, Germany), serum HDL cholesterol (enzymatic method, Roche Diagnostics, Mannheim, Germany), and serum fasting glucose (hexokinase, Olympus System Reagent, Germany) concentration were determined from frozen (−70 °C) serum samples taken during the health examination. We used the threshold values of the International Diabetes Federation (IDF) for the metabolic syndrome [39] in order to categorize these variables, as follows: For serum triglycerides (mmol/L) <1.7 and ≥1.7, for serum HDL cholesterol (mmol/L) ≥1.03 in men and ≥1.29 in women and <1.03 in men and <1.29 in women, and for fasting glucose (mmol/L) <5.6 and ≥5.6.

Blood pressure was measured at the health examination with a standard mercury manometer (Mercuro 300, Speidel & Keller, Jungingen, Germany) twice, with a two-minute interval, and the mean of the two measurements was calculated. The information on the use of antihypertensive medication was drawn from the interview. Elevated blood pressure was determined according to the IDF’s definition [39], as follows: Systolic pressure ≥130 mmHg or diastolic pressure ≥85 mmHg, or the use of antihypertensive medication.

Information for the T2D (ICD-10, E11) variable was assembled from questionnaires, an interview, the health examination, and a nationwide register of patients receiving diabetes medication reimbursement that is kept by the Social Insurance Institution. The registers were linked to the study population by the unique social security numbers of each Finnish citizen. Osteoarthritis in the knee and hip joints was diagnosed by trained physicians (who worked according to written instructions and applied preset criteria) at the health examination on the basis of standardized physical status, symptoms, and medical history [32,40].

#### 2.2.5. Mental Health

Depressive and anxiety disorders were diagnosed using the German Composite International Diagnostic Interview (M-CIDI) and the DSM-IV diagnostics [41]. Concerns about one’s appearance and concerns about one’s health were measured by two items on the self-administered Beck Depression Inventory (BDI) [42]. Sense of coherence (SOC, i.e., a disposition to consider life as comprehensible, manageable, and meaningful) was assessed using the self-administered multidimensional coping inventory—the SOC-13 scale [43]. Social support received from people close to oneself was measured with a self-administered scale on a questionnaire.

### 2.3. Statistical Methods

The linear and logistic models were used to determine the strength of association between the potential determinants and the two outcome variables (i.e., self-report dieting attempts and previous IWL). The effect size for the independent variables was estimated as the model-adjusted mean [44] in the categories of variables in the linear model and as the relative odds in the logistic model.

Men and women were analyzed separately and combined and the potential determinants were grouped into four domains, as follows: Socio-demographic, lifestyle, somatic health, and psychiatric and psychological factors. Two models were used. Model 1 included sex and age. Model 2 (the full model) included sex (only when men and women were analyzed combined), age, education, BMI, FFMI, leisure-time physical activity, sitting time, smoking, energy intake, AHEI, daily consumption of certain sugary products, sleep duration, serum triglycerides, serum HDL cholesterol, blood pressure, T2D, osteoarthritis, SOC, concerns about one’s appearance, and concerns about one’s health. The full model was evaluated in separate domain-specific analyses and the variables were collected, excluding those which were not significant (e.g., marital status, residential area, alcohol consumption, depressive disorder, anxiety disorder, and social support received from people close to oneself) or were illogical (serum fasting glucose) in the domain-specific analyses.

In addition to BMI and FFMI, other measures of body composition (e.g., waist circumference (cm) and fat mass index (fat mass kg/m^2^)) were considered to be included as determinants. However, due to high correlations between them and BMI and as their associations with the dieting variables were nearly similar to those of BMI, these measures were excluded from this study and BMI was chosen to represent such obesity measures. FFMI was chosen to be included as its associations with the dieting variables differed more distinctly from corresponding associations of the other body composition measures.

The possible effect modification of sex or BMI was studied by including an interaction term in the model, between the respective variable and the potential determinants.

Due to the numerous analyses in this study, we performed a Bonferroni correction, which attenuated part of the associations to be non-significant. However, when performing a Bonferroni correction, the possibility of rejecting true positive findings grows and, as the findings met our initial hypotheses, we chose to approve the found results.

All analyses were conducted using SAS 9.3 [45].

## 3. Results

Of the men, 24% had attempted to diet and 10% had intentionally lost weight during the previous year, whereas of the women, as expected, the corresponding numbers were higher—39% and 15% (Table 1). The mean age of the study population was 47.9 years and approximately one third of them were highly educated. The mean BMI was 26.8 kg/m^2^. Roughly one-fifth participated in regular vigorous training and one-third were current smokers. In all, 27% of the men reported daily consumption of certain sugary products, while the corresponding value for the women was 19%. Of the men, 11% were concerned about their appearance and 28% about their health and of the women, the values were 22% and 33%, respectively.

### 3.1. Dieting Attempts

#### 3.1.1. Sociodemographic Factors and Dieting Attempts

Dieting attempts and age had a statistically significant inverse gradient, with relative odds of 0.63 (95% CI 0.41–0.99) in men and 0.37 (95% CI 0.25–0.55) in women between individuals 60–69 and 30–39 years old (*p*-value for sex interaction = 0.02) (Table 2). Higher education was related to dieting attempts, whereas marital status, the number of children, and residential area did not show any association.

#### 3.1.2. Lifestyle and Dieting Attempts

In line with the previous findings, dieting attempts were strongly dependent on BMI, especially in men (*p*-value for sex interaction <0.001) (Table 2). The relative odds of dieting attempts between individuals with obesity and individuals with normal weight in the multivariate model were 9.54 (95% CI 5.33–17.1) in men and 2.81 (95% CI 1.83–4.31) in women. Moreover, the relative odds of dieting attempts were elevated in the three middle quintiles of the FFMI, compared to the lowest quintile. Dieting attempts were also frequent in individuals who were physically more active during their leisure time (for regular vigorous training vs. low activity: OR = 1.65, 95% CI 1.29–2.09) and in individuals sitting more during the day. Former smokers were more commonly dieters than those who have never smoked or current smokers. The AHEI showed a statistically significant positive gradient with dieting attempts, whereas energy intake and the daily consumption of certain sugary products showed an inverse association. Short sleep duration was associated with dieting attempts in men. No association between dieting attempts and alcohol consumption was found.

BMI and smoking had a suggestive interaction (*p*-value for interaction = 0.12), the relative odds of dieting attempts for ex-smokers with obesity compared to never-smokers with obesity being 1.85 (95% CI 1.27–2.70), whereas no similar association was seen in the other BMI groups (Table S1). Individuals within the highest energy intake quintile had lower odds of having made dieting attempts compared to those in the lowest intake quintile among those with normal weight and those with overweight (OR = 0.43, 95% CI 0.26–0.69 and OR = 0.56, 95% CI 0.39–0.79, respectively), whereas in those with obesity, no such association was observed (*p*-value for interaction = 0.02). Moreover, the daily consumption of certain sugary products had a statistically significant interaction with BMI (*p* = 0.002). While individuals with normal weight or obesity did not show significant differences between consumption groups, those who had overweight and consuming sugary products daily had lowered odds of dieting attempts compared to those who hadoverweight and not consuming sugary products daily (OR 0.53, 95% CI 0.40–0.72).

#### 3.1.3. Somatic Health and Dieting Attempts

The study of somatic health-related factors (after age adjustment) relatively consistently showed dieting attempts to be statistically significantly more frequent in persons with symptoms of metabolic syndrome or a diagnosed disease (Table 2). After the inclusion of all variables in the multivariate model, men with T2D had significantly greater odds of making dieting attempts compared to those without T2D (OR = 2.13, 95% CI 1.20–3.78); whereas in women, having T2D showed no association with dieting attempts (*p*-value for sex interaction = 0.02). On the contrary, despite the lack of significant sex interaction in the multivariate model, the associations between dieting attempts and elevated serum triglycerides and lowered serum HDL remained significant, principally in women.

Generally, no significant interactions appeared between BMI and the indicators of somatic health considered in the prediction of dieting attempts (Table S1). The only exception was knee or hip osteoarthritis (*p*-value for BMI interaction = 0.03), which showed suggestive elevated relative odds (1.65, 95% CI 0.99–2.77) between the subjects with and without it in the group with overweight.

#### 3.1.4. Mental Health and Dieting Attempts

Neither diagnosed depressive or anxiety disorders nor social support received from people close to oneself were significantly related to dieting attempts. In terms of SOC, however, the frequency of dieting attempts was higher with lower SOC levels (OR = 1.45, 95% CI 1.15–1.82 between the lowest and the highest quartile) (Table 2). Moreover, dieting attempts were related to concerns about one’s appearance and one’s health. Despite the lack of significant sex interactions, the relation to concerns for one’s health was pronounced in men (OR = 1.37, 95% CI 1.04–1.81).

No significant interactions appeared between BMI and psychiatric or psychological factors when predicting dieting attempts (Table S1). However, BMI and concerns about one’s appearance showed a non-significant suggestive interaction, according to which the concerns seemed to be more strongly associated with dieting attempts in individuals with normal weight or withoverweight than in individuals with obesity.

### 3.2. IWL

#### 3.2.1. Sociodemographic Factors and IWL

In the multivariate model, previous IWL was statistically significantly associated with younger age with a relative odds of 0.42 (95% CI 0.29–0.62) between individuals aged 60–69 and 30–39. The association was more pronounced in women (*p*-value for sex interaction = 0.05) (Table 3). Higher education was related to IWL, whereas no associations appeared for any of the other sociodemographic variables considered.

In the interaction analyses of BMI and sociodemographic factors, BMI and sex showed a significant interaction (*p* = 0.01) (Table S1). In those with normal weight, women had nearly triple the odds of having intentionally lost weight compared to men (OR = 2.92, 95% CI 1.80–4.74), whereas in those with obesity there were no significant differences between men and women.

#### 3.2.2. Lifestyle and IWL

A suggestive association emerged between BMI and IWL due to a strong association among men. The relative odds between men with obesity and men with normal weight was 4.44 (95% CI 2.03–9.67, *p*-value for sex interaction = 0.01) (Table 3). In women, no differences occurred between the pre-defined BMI groups. Moreover, IWL was unusual in individuals with the lowest FFMI values. Of the other lifestyle-related variables, moderate leisure-time physical activity, long sitting time, and smoking (both currently and formerly) were related to IWL. In addition, IWL was associated with having a higher quality of diet, lower energy intake and not consuming daily certain sugary products. Moreover, short sleep duration was associated with IWL in men.

No significant interaction appeared between BMI and the lifestyle-related factors (Table S1). However, BMI and energy intake showed a tendency for an interaction (*p* = 0.15). Energy intake was only significantly inversely associated with IWL in individuals with normal weight.

#### 3.2.3. Somatic Health and IWL

IWL was associated with low serum HDL cholesterol (OR = 1.33, 95% CI 1.07–1.65 between low and normal concentration) (Table 3). In men, those having T2D or knee or hip osteoarthritis had more commonly intentionally lost weight, the relative odds being 2.16 (95% CI 1.13–4.15) for T2D and 2.27 (95% CI 1.21–4.29) for osteoarthritis. In general, no significant interaction appeared between BMI and the indicators of somatic health that were considered (Table S1). The only exception was osteoarthritis (*p*-value for interaction = 0.01), which showed a strong positive association in individuals who had overweight (OR = 2.74, 95% CI 1.51–4.97).

#### 3.2.4. Mental Health and IWL

IWL was associated with none of the psychiatric or psychological factors in the additive model. There was, however, a significant interaction for concern about one’s appearance and BMI (*p* = 0.02) (Table S1). Individuals with normal weight and concerns about their appearance had more frequently intentionally lost weight compared to those without concerns about their appearance (OR = 1.71, 95% CI 1.07–2.72), while no differences appeared in those with obesity or with overweight.

## 4. Discussion

We found dieting attempts and IWL to be more common in younger age groups, particularly in women, which is in line with previous findings [14,15,16,17,18,21,46,47,48,49,50,51]. Both younger individuals and women, in particular, are affected by social pressure and the desire to be lean [52]. Dieting attempts and IWL being more frequent in those with higher education is backed up in the previous literature [14,15,17,19,20,21,29,31,47,53,54]. Education increases knowledge on the harmful consequences of obesity, which might push more highly educated individuals to try to lose weight more often. Moreover, it is possible that social pressure to be lean is more prevalent among those with higher education.

Individuals within the three middle FFMI quintiles had attempted dieting more often than those within the lowest or the highest quintiles. The lack of association between the highest and lowest quintiles derived from the presence of BMI in the multivariate model, suggesting that high FFMI in the absence of obesity is not associated with dieting. Loss of muscle mass has been linked to impaired functional capacity and mortality [55], hence the need or resources to attempt dieting may be absent in the lives of those with low FFMI values. Alternatively, it is possible that measuring fat free mass with bioimpedance may not be accurate for individuals with extreme BMI values or with abnormal hydration [56]. In the sex-specific analysis, women with the highest FFMI had intentionally lost weight over twice as often as women with the lowest FFMI. The FFMI measures the amount of fat free mass relative to the person’s height [57]. It may be that a higher FFMI is a result of intentionally losing weight and, expressly, fat mass. Alternatively, it is possible that those in the highest quintile are more often athletes and, particularly in women, feel the need to lose weight in order to stay in good shape. Surprisingly, even though women with the highest FFMI had most frequently lost weight intentionally, the same group of women did not differ from those with the lowest FFMI with regard to dieting attempts. Indeed, it seems that women with the highest FFMI did not attempt dieting any more often than women with lower values; but when they did so, they more commonly succeeded.

Our finding on the positive association between BMI and dieting attempts is in line with previous results [5,14,15,16,17,18,20,21,26,29,46,47,48,49,51,58,59,60,61,62,63,64,65]. People with obesity may have health-related reasons and other personal reasons to attempt to lose weight, but they may also have more social pressure to report dieting even though they have not necessarily dieted. Even though women within each BMI category reported dieting attempts more often than men, the difference between sexes was more pronounced at lower BMI levels, which is consistent with the previous literature [15,16,17,19,20,48]. In modern societies, women seem to have stricter social ideal weight norms and diet when they have normal weight, whereas the authors speculate that men start dieting when they actually become affected by overweight or obesity. After controlling for potential confounding factors, IWL was only related to BMI in men. Apparently, men with obesity take weight-loss efforts more seriously, while for women, a higher BMI does not make a difference to the successfulness of dieting attempts.

As far as we know, our finding on more sitting time being positively associated with dieting attempts and IWL was the first on this topic. Obesity, however, has been found to be associated with more sitting time [66].

Physical activity was positively associated with dieting attempts, which is in line with most [21,22,23,24,25,67] but not all previous results [58]. Physical activity may be a strategy to attempt to lose weight [12] or it may be that, in the vigorous physical activity category, there are more athletes who take part in competitive sports. Such athletes may diet to stay in shape and for optimal performance. Alternatively, it is also possible that those who feel pressure to report dieting (those with obesity) also feel pressure to report vigorous physical activity while not actually dieting or exercising.

Dieting attempts and IWL were more prevalent in former smokers than in those who had never smoked, especially in women and in individuals with obesity. However, even though there are some contrary findings on former smoking in men [17], some studies support these results [14,16]. Former smokers may have gained weight after quitting smoking and consequently try to lose weight [68]. Alternatively, former smokers may have made a lifestyle change that includes both quitting smoking and dieting to lose weight.

Our findings are in line with the results of previous studies regarding the inverse associations between energy intake and dieting attempts and IWL [27,29,30,31,38,69], the direct association between quality of diet (or in previous studies, the components of a healthy diet) and dieting attempts and IWL [24,26,28,31,38,69], and the inverse associations between the daily consumption of certain sugary products and dieting attempts and IWL [22,24,28,64,69]. This is presumably due to using dieting (both decreasing energy intake and altering one’s diet to be healthier) as a strategy to lose weight [12]. Dieters, moreover, have been shown to under-report their energy intake (especially their intake of sugary and fatty foods) and over-report their intake of food items considered healthy and socially desirable (e.g., vegetables), which may lead to biased results [70]. The importance of dietary habits is crucial in the development of obesity and numerous diseases. Thus, the role of a healthy diet, and not just counting calories, is essential when trying to prevent chronic diseases [71].

When looking into associations between energy intake and dieting attempts in BMI categories, significant inverse associations only emerged in those with normal weight or those who had overweight, while in those with obesity, the association remained non-existent. This is contrary to the finding by Neumark-Sztainer et al. [54], according to which a significant inverse association only emerged among those who had overweight, but not among those with normal weight. In men only, those sleeping ≤6 h a night had attempted dieting and had had IWL more often than those sleeping 7 to 8 h a night. In accordance, a Canadian study showed a short sleep duration to be associated with dieting attempts and previous weight loss [72]. A short sleep duration has also been linked to obesity and findings even suggest that it acts as an obstacle to weight loss [73]. Hence, those sleeping inadequately may attempt dieting and have IWL due to short sleep-related weight gain. However, the association remained significant even after adjusting for BMI. Thus, it is possible that some other reasons lie behind the association.

No previous studies exist on the association between serum triglycerides and dieting attempts at the population level. One study, however, demonstrated that young women with high dietary restraint had higher serum triglycerides than those with low dietary restraint [74]. In addition, high serum cholesterol has been associated with dieting attempts [18,58]. Moreover, weight cycling, in most cases following repeated dieting periods, has been found to be associated with elevated serum lipids [75]. Indeed, weight regain after weight loss may elevate levels beyond their initial value. Hence, it is possible that the positive association found in our study, primarily in women, derives from previous dieting attempts and weight regain. Alternatively, awareness of disadvantageous values may drive individuals to try to lose weight. Additionally, these possible reasons may also hold true for the inverse associations found between HDL and dieting attempts and IWL, maybe more strongly in women.

T2D was only associated with dieting attempts and IWL in men. Findings from an American study support this association, although in that study the connection was found in both sexes [58]. However, in a Canadian study, T2D was found to be unrelated to dieting attempts [23]. The association between knee or hip osteoarthritis and dieting attempts and IWL at the population level has been, prior to the present study, an unexamined field, although weight control should be an essential part of treatment. We found men with osteoarthritis to have had an IWL more often than men without the disease. In a BMI-specific analysis, the association was only significant in those who had overweight. It may be that these individuals previously had obesity but had succeeded in losing weight, whereas those with current obesity lack the association, due to not having succeeded. However, a suggestive association between osteoarthritis and also dieting attempts emerged in those who were overweight but not in those with obesity, which does not affirm the preceding speculation; and BMI-specific associations may also arise from small n-sizes in these categories in a population-based study. In our study, men with T2D or an osteoarthritis diagnosis may have taken the diagnosis as a serious warning sign to start losing weight in order to stop the progression of the disease. Women with the diagnosis may act alike; but as women already diet more often than men, the difference between the groups remains non-significant.

Our inverse finding between SOC and dieting attempts is the first on this topic. Individuals with low SOC values (i.e., those with a relatively poor capacity to cope with everyday life and to manage its stressors [43]) have previously been shown to be less successful in achieving health-related lifestyle changes and a better quality of life than persons with higher SOC values [76]. Thus, they may be prone to misperceiving their weight as a problematic issue, possibly due to their lack of resources to deal with other life issues perceived as too difficult to manage. Thus a dieting attempt may be used as a means to seek better overall control of one’s life instead of being content with oneself or focusing on making other attempts to change their negative dispositions. Alternatively, greater attempted dieting in persons with a low SOC might be seen as a sign of persons becoming aware that something in their lives needs to be changed.

Most of the reported motives that push people to attempt dieting are related to appearance and health reasons [12]. In this study, the determinants studied were not asked about as motives or strategies for dieting but as independent factors, so it is impossible to specify whether the determinants are actually reasons for dieting or only associated with them. However, it can be speculated that individuals reporting concerns about their appearance or health attempt dieting because of these factors. Those having concerns about their appearance or health reported more dieting attempts but not more IWL. Such concerns may be a reason for dieting, but as they are only associated with dieting attempts and not with weight loss, they may not be such a strong reason to actually lose weight. The association between concerns for one’s appearance and dieting attempts was slightly pronounced in women, whereas the association between concerns for one’s health and dieting attempts was more pronounced in men. Such a tendency is in line with men with a disease diagnosis (T2D or osteoarthritis) dieting more commonly. In a study conducted with women, those trying to lose weight and motivated by appearance reasons used more unhealthy dieting strategies (e.g., skipping meals, eating only one type of food, vomiting, using laxatives and diuretics) and also reported more lapses, whereas those motivated by health reasons used healthier strategies [77]. When looking into associations between concerns about one’s appearance and dieting attempts in BMI categories, the association only appeared in those with normal weight and those who had overweight. It might be that appearance concerns are more present in the lives of those with lower BMI, while, along with a higher BMI, health-related reasons become more relevant.

The present study contains some major strengths, as follows: The large representative adult population sample; a comprehensive set of potential determinants covering demographic, lifestyle, somatic health, and psychiatric and psychological factors; the availability of body composition-related measures and biomarkers; and the simultaneous exploration of dieting attempts and IWL.

However, there are also some limitations. First, because of the cross-sectional study design, it remains unresolved whether dieting attempts and IWL are a cause or a consequence of the determinants or are associated with the determinants for some other reason. Second, as the dieting variables were self-reported, the concept, seriousness, and continuity of dieting may vary between individuals, making the dieters’ group heterogeneous. That may affect the interpretation of the results. Third, we included all individuals attempting dieting with previous weight loss in the IWL group, hence the range of self-reported weight loss is quite extensive (range 1–38 kg, mean 5.43 (SD 4.21) kg). Fourth, we did not exclude individuals with cancer, T2D, or any other disease from the sample, although these individuals may have attempted dieting and experienced unintentional weight loss. Fifth, the small number of subjects in the categories of certain determinants, including somatic diseases and psychiatric disorders, makes the distributions of these variables skewed; thus, possibly masking associations. Sixth, even though we included a vast set of possible determinants in our scrutiny, other important determinants may be missing. Seventh, the inclusion of all potential determinants in the final models may have caused overadjustment. Finally, due to the numerous analyses conducted, the possibility of false positive findings cannot be ruled out.

## 5. Conclusions

This study was among the first to concentrate on a comprehensive scrutiny of the determinants of self-report dieting attempts and IWL. Dieting attempts were common in women within every BMI category; whereas in men with normal weight, they were relatively infrequent. Moreover, the prevalence of IWL grew along with BMI in men but not in women. It seems that in women, dieting behaviour is not that dependent on a real need to lose weight, while it seems that men do not start dieting until they become affected by overweight or develop an obesity-related disease (such as T2D or osteoarthritis).

The exploration of the determinants associated with dieting attempts and IWL is important in regard to their use as confounding factors when analyzing the associations between dieting attempts and the incidence of chronic diseases. Moreover, these findings can be used for determining subgroups with obesity that would benefit from weight loss but abstain from dieting. Simultaneously, subpopulations with normal weight attempting dieting can be revealed, which is important in order to plan preventive actions against unnecessary dieting attempts and possible future weight gain. Further studies and meta-analyses, in particular, are needed to strengthen the information on the determinants of dieting attempts and IWL.

## Figures and Tables

**Table 1:** Characteristics of the men and women in the study population.

	Men and Women (*n* = 4525)		Men (*n* = 2147)		Women (*n* = 2378)	
Determinants	*n*	Mean (SD) or %	*n*	Mean (SD) or %	*n*	Mean (SD) or %
**Dieting**						
Dieting attempts * (%)	4525	31.8	2147	24.2	2378	38.6
IWL * (%)	4525	13.0	2147	10.4	2378	15.3
**Socio-demographic factors**						
Age (years)	4525	47.9 (10.7)	2147	47.7 (10.6)	2378	48.0 (10.8)
High education (%)	4513	32.4	2141	26.6	2372	37.6
**Lifestyle related factors**						
BMI (kg/m^2^)	4525	26.8 (4.68)	2147	27.1 (4.12)	2378	26.5 (5.11)
FFMI (fat free mass kg/m^2^)	4382	19.3 (2.41)	2095	21.0 (1.90)	2287	17.8 (1.70)
Regular vigorous training (%)	4498	19.1	2133	22.2	2365	16.2
Sitting time (min/day)	4363	340 (169)	2082	340 (176)	2281	340 (162)
Current smoking (%)	4510	30.2	2140	35.5	2370	25.3
Energy intake (kcal/day)	4221	2304 (791)	1975	2408 (827)	2246	2213 (746)
AHEI (score) (range 7–35)	4221	21.2 (4.94)	1975	21.1 (4.96)	2246	21.3 (4.91)
Daily consuming sweets, chocolate, cookies, dried fruits or sugar-sweetened drinks (%)	4513	22.5	2143	26.7	2370	18.7
Sleep duration (hours)	4224	7.46 (1.04)	1981	7.33 (1.02)	2243	7.57 (1.05)
**Somatic health**						
Fs-triglycerides (mmol/L)	4510	1.57 (1.06)	2142	1.83 (1.31)	2368	1.33 (0.69)
Fs-HDL (mmol/L)	4510	1.34 (0.38)	2142	1.21 (0.33)	2368	1.45 (0.38)
Elevated blood pressure ^†^ (%)	4525	55.9	2147	64.4	2378	48.2
T2D (%)	4525	3.54	2147	3.87	2378	3.24
Osteoarthritis (%)	4467	4.88	2131	5.21	2336	4.58
**Psychological factors**						
SOC (mean score) (range 1–7)	4314	5.48 (0.80)	2023	5.50 (0.81)	2291	5.46 (0.80)
Concerns about one’s appearance (%)	4471	16.6	2127	11.1	2344	21.6
Concerns about one’s health (%)	4480	30.9	2125	28.1	2355	33.3

*n*, Number of subjects in respective category; SD, Standard deviation; IWL, Intentional weight loss; BMI, Body mass index; FFMI, Fat free mass index; AHEI, Alternate Healthy Eating Index; Fs-, Fasting serum; HDL, High density lipoprotein; T2D, Type 2 diabetes; SOC, Sense of coherence. * During the previous year. ^†^ Systolic blood pressure ≥130 mmHg or diastolic blood pressure ≥85 mmHg, or use of antihypertensive medication.

**Table 2:** Self-report dieting attempts during the previous year in men and women by potential determinants (*n* = 4525).

	Men and Women (*n* = 4525)					Men (*n* = 2147)					Women (*n* = 2378)					*p* for Inter-Action by Sex
	Age and Sex-Adjusted		Full Model *			Age-Adjusted		Full Model *			Age-Adjusted		Full Model *			
Determinants	*n*	(%)	*n*	OR	95% CI	*n*	(%)	*n*	OR	95% CI	*n*	(%)	*n*	OR	95% CI	
**Socio-demographic factors**																
Age (years)	4525		3749			2147		1780			2378		1969			**0.02**
30–39	1227	31.6	1055	1		583	21.4	489	1		644	40.7	566	1		
40–49	1327	33.8	1119	0.83	0.68–1.02	629	26.9	535	1.07	0.77–1.49	698	40.1	584	**0.70**	**0.54–0.92**	
50–59	1176	32.9	970	**0.58**	**0.46–0.72**	574	24.7	475	**0.63**	**0.43–0.90**	602	40.4	495	**0.54**	**0.40–0.74**	
60–69	795	27.0	605	**0.45**	**0.34–0.60**	361	23.3	281	**0.63**	**0.41–0.99**	434	30.6	324	**0.37**	**0.25–0.55**	
*p* for trend ^†^		0.14					0.40					**0.009**				
Education (%)	4513		3749			2141		1780			2372		1969			0.37
Low	1450	29.8	1108	1		691	22.1	530	1		759	36.9	578	1		
Intermediate	1603	30.9	1366	1.10	0.90–1.35	881	23.1	753	1.10	0.80–1.50	722	38.3	613	1.13	0.86–1.48	
High	1460	34.8	1275	**1.39**	**1.12–1.72**	569	28.5	497	**1.63**	**1.15–2.31**	891	40.4	778	1.26	0.96–1.65	
*p* for heterogeneity		**0.01**					**0.02**					0.39				
**Lifestyle related factors**																
BMI (kg/m^2^)	4525		3749			2147		1780			2378		1969			**<0.0001**
<25	1746	14.8	1455	1		692	6.28	569	1		1054	22.2	886	1		
25–29.9	1805	36.2	1517	**2.52**	**1.99–3.19**	1010	26.2	854	**4.17**	**2.69–6.47**	795	45.7	663	**2.20**	**1.64–2.95**	
≥30	974	54.1	777	**4.24**	**3.03–5.93**	445	47.6	357	**9.54**	**5.33–17.1**	529	60.5	420	**2.81**	**1.83–4.31**	
*p* for trend ^†^		**<0.0001**					**<0.0001**					**<0.0001**				
FFMI quintiles ^‡^ (fat free mass kg/m^2^)	4382		3749			2095		1780			2287		1969			**0.001**
1st (lowest)	875	12.8	752	1		419	6.24	344	1		456	18.3	408	1		
2nd	877	24.1	745	**1.54**	**1.16–2.05**	419	17.9	357	**1.95**	**1.15–3.31**	458	29.5	388	**1.43**	**1.01–2.02**	
3rd	876	32.0	754	**1.55**	**1.16–2.08**	419	21.9	369	1.66	0.97–2.86	457	41.3	385	**1.60**	**1.11–2.30**	
4th	876	40.1	753	**1.64**	**1.18–2.26**	419	31.7	361	**2.03**	**1.14–3.60**	457	48.0	392	1.45	0.97–2.17	
5th	878	50.4	745	1.08	0.72–1.62	419	43.5	349	1.49	0.76–2.92	459	57.4	396	0.82	0.48–1.40	
*p* for trend		**<0.0001**					**<0.0001**					**<0.0001**				
Leisure-time physical activity	4498		3749			2133		1780			2365		1969			0.38
Low	1108	29.4	900	1		543	21.3	439	1		565	36.9	461	1		
Moderate	2533	31.9	2097	**1.40**	**1.15–1.70**	1117	25.1	919	**1.61**	**1.17–2.22**	1416	38.1	1178	1.28	0.99–1.64	
Regular vigorous training	857	35.2	752	**1.65**	**1.29–2.09**	473	25.9	422	**1.70**	**1.18–2.47**	384	43.9	330	**1.69**	**1.22–2.34**	
*p* for heterogeneity		**0.02**					0.16					0.07				
Sitting time tertiles ^§^ (min/day)	4363		3749			2082		1780			2281		1969			0.45
1st (lowest)	1444	29.2	1211	1		677	22.0	557	1		767	35.7	654	1		
2nd	1418	30.5	1225	1.06	0.88–1.28	693	22.7	604	1.12	0.82–1.52	725	37.7	621	1.07	0.83–1.37	
3rd	1501	35.8	1313	**1.30**	**1.08–1.57**	712	28.1	619	**1.48**	**1.09–2.02**	789	42.7	694	1.20	0.94–1.53	
*p* for trend		**0.0001**					**0.008**					**0.004**				
Smoking	4510		3749			2140		1780			2370		1969			0.60
Never	2207	31.2	1860	1		779	23.3	663	1		1428	38.5	1197	1		
Former smoker	943	39.7	788	**1.28**	**1.05–1.56**	601	30.5	501	1.06	0.78–1.43	342	48.0	287	**1.42**	**1.07–1.89**	
Current smoker	1360	27.2	1101	0.88	0.73–1.07	760	19.9	616	0.83	0.61–1.12	600	33.6	485	0.88	0.69–1.13	
*p* for heterogeneity		**<0.0001**					**<0.0001**					**<0.0001**				
Energy intake quintiles ^‖^ (kcal/day)	4221		3749			1975		1780			2246		1969			0.26
1st (lowest)	844	37.0	735	1		395	28.8	351	1		449	44.0	384	1		
2nd	844	33.3	755	0.86	0.68–1.09	395	28.6	358	1.15	0.80–1.65	449	37.4	397	**0.68**	**0.50–0.94**	
3rd	844	30.1	743	**0.74**	**0.58–0.94**	395	21.8	349	0.75	0.51–1.10	449	37.4	394	0.73	0.53–1.00	
4th	844	30.9	758	**0.76**	**0.60–0.96**	395	23.3	357	0.79	0.54–1.16	449	37.6	401	0.74	0.54–1.01	
5th	845	28.9	758	**0.60**	**0.47–0.77**	395	20.3	365	**0.62**	**0.42–0.92**	450	36.7	393	**0.59**	**0.43–0.83**	
*p* for trend		**0.0002**					**0.001**					**0.05**				
AHEI quintiles ^¶^	4221		3749			1975		1780			2246		1969			0.67
1st (lowest)	759	22.9	653	1		373	15.8	323	1		386	29.2	330	1		
2nd	835	27.6	742	**1.37**	**1.05–1.78**	390	20.0	354	1.37	0.89–2.11	445	34.4	388	1.35	0.96–1.89	
3rd	970	31.4	870	**1.45**	**1.13–1.88**	436	25.2	393	**1.70**	**1.12–2.58**	534	36.9	477	1.32	0.95–1.83	
4th	787	34.9	704	**1.70**	**1.31–2.23**	369	27.4	338	**1.80**	**1.17–2.77**	418	41.5	366	**1.60**	**1.13–2.26**	
5th	870	42.2	780	**2.26**	**1.74–2.95**	407	33.7	372	**2.18**	**1.43–3.33**	463	49.8	408	**2.29**	**1.62–3.23**	
*p* for trend		**<0.0001**					**<0.0001**					**<0.0001**				
Daily consuming certain sugary products **	4513		3749			2143		1780			2370		1969			0.23
No	3499	33.9	2925	1		1571	26.1	1310	1		1928	41.0	1615	1		
Yes	1014	24.2	824	**0.73**	**0.60–0.89**	572	18.9	470	0.85	0.63–1.13	442	28.4	354	**0.61**	**0.46–0.81**	
*p* for heterogeneity		**<0.0001**					**0.0007**					**<0.0001**				
Sleep duration (hours)	4224		3749			1981		1780			2243		1969			**0.01**
≤6	583	34.6	491	1		316	30.7	266	1		267	37.5	225	1		
7–8	3170	31.8	2866	0.84	0.67–1.05	1509	23.3	1381	**0.60**	**0.44–0.83**	1661	39.3	1485	1.13	0.82–1.56	
≥9	471	30.9	392	0.78	0.57–1.07	156	25.5	133	0.72	0.43–1.22	315	36.0	259	0.94	0.62–1.42	
*p* for heterogeneity		0.34					**0.02**					0.50				
**Somatic health**																
Fs-triglycerides (mmol/L)	4510		3749			2142		1780			2368		1969			0.59
<1.7	3095	27.9	2584	1		1267	20.0	1044	1		1828	35.1	1540	1		
≥1.7	1415	40.2	1165	**1.20**	**1.00–1.44**	875	30.4	736	1.05	0.81–1.37	540	50.5	429	**1.40**	**1.07–1.82**	
*p* for heterogeneity		**<0.0001**					**<0.0001**					**<0.0001**				
Fs-HDL (mmol/L)	4510		3749			2142		1780			2368		1969			0.56
≥1.03 in men or ≥1.29 in women	2968	27.8	2478	1		1477	21.2	1235	1		1491	33.8	1243	1		
<1.03 in men or <1.29 in women	1542	39.3	1271	1.18	0.99–1.40	665	30.9	545	1.02	0.77–1.35	877	46.7	726	**1.27**	**1.02–1.58**	
*p* for heterogeneity		**<0.0001**					**<0.0001**					**<0.0001**				
Elevated blood pressure ^††^	4525		3749			2147		1780			2378		1969			0.12
No	1995	27.5	1691	1		764	19.9	642	1		1231	34.1	1049	1		
Yes	2530	35.1	2058	0.97	0.82–1.16	1383	26.6	1138	0.98	0.75–1.29	1147	43.4	920	0.99	0.78–1.24	
*p* for heterogeneity		**<0.0001**					**0.0007**					**<0.0001**				
T2D	4525		3749			2147		1780			2378		1969			**0.02**
No	4365	31.1	3633	1		2064	23.3	1715	1		2301	38.1	1918	1		
Yes	160	51.1	116	1.43	0.94–2.19	83	46.9	65	**2.13**	**1.20–3.78**	77	54.2	51	0.86	0.46–1.61	
*p* for heterogeneity		**<0.0001**					**<0.0001**					**0.005**				
Knee or hip osteoarthritis	4467		3749			2131		1780			2336		1969			0.26
No	4249	31.3	3582	1		2020	23.5	1702	1		2229	38.3	1880	1		
Yes	218	41.4	167	0.92	0.63–1.34	111	34.9	78	0.96	0.54–1.69	107	47.0	89	0.89	0.54–1.45	
*p* for heterogeneity		**0.002**					**0.008**					0.08				
**Psychological factors**																
SOC quartiles ^‡‡^	4314		3749			2023		1780			2291		1969			0.79
1st (highest)	1133	28.8	1003	1		543	23.4	500	1		590	33.4	503	1		
2nd	1127	30.5	1007	0.99	0.80–1.22	558	22.6	502	0.95	0.68–1.32	569	37.7	505	1.07	0.81–1.41	
3rd	1045	33.5	923	1.07	0.86–1.33	428	25.9	377	0.95	0.66–1.35	617	40.1	546	1.18	0.89–1.55	
4th (lowest)	1009	36.8	816	**1.45**	**1.15–1.82**	494	27.7	401	1.40	0.98–1.99	515	45.0	415	**1.47**	**1.08–2.00**	
*p* for trend		**<0.0001**					**0.06**					**<0.0001**				
Concerns about one’s appearance	4471		3749			2127		1780			2344		1969			0.59
Does not feel that looks any worse than used to	3729	30.3	3144	1		1891	23.1	1603	1		1838	36.8	1541	1		
Concerns about one’s appearance	742	40.1	605	**1.27**	**1.03–1.58**	236	33.4	177	1.29	0.87–1.93	506	46.2	428	**1.29**	**1.00–1.66**	
*p* for heterogeneity		**<0.0001**					**0.0006**					**0.0001**				
Concerns about one’s health	4480		3749			2125		1780			2355		1969			0.18
Not worried about their health more than usually	3097	29.1	2617	1		1527	21.7	1296	1		1570	35.8	1321	1		
Concerns about one’s health	1383	38.2	1132	**1.21**	**1.02–1.43**	598	30.7	484	**1.37**	**1.04–1.81**	785	44.9	648	1.17	0.94–1.47	
*p* for heterogeneity		**<0.0001**					**<0.0001**					**<0.0001**				

*n*, Number of subjects in respective category; OR, Odds ratio; CI, Confidence interval; BMI, Body mass index; FFMI, Fat free mass index; AHEI, Alternate Healthy Eating Index; Fs-, Fasting serum; HDL, High density lipoprotein; T2D, Type 2 diabetes; SOC, Sense of coherence. Bolded results are statistically significant. * Adjusted for all the other variables in the table: sex (only when men and women analyzed together), age (continuous), education, BMI (continuous), FFMI (continuous as quintiles), leisure-time physical activity, sitting time (continuous as tertiles), smoking, energy intake (continuous as quintiles), AHEI (continuous as quintiles), daily consuming certain sugary products, sleep duration, fs-triglycerides, fs-HDL, elevated blood pressure, T2D, osteoarthritis, SOC (continuous as quartiles), concerns about one’s appearance, and concerns about one’s health. ^†^ Trend for continuous variable. ^‡^ FFMI quintile ranges (fat free mass kg/m^2^): 1st 14.3–19.5 for male, 11.1–16.3 for female; 2nd 19.6–20.5 for male, 16.4–17.2 for female; 3rd 20.6–21.4 for male, 17.3–18.1 for female; 4th 21.5–22.6 for male, 18.2–19.2 for female; 5th 22.7–29.4 for male, 19.3–24.2 for female. ^§^ Sitting time tertile ranges (min): 1st 0–236 for male, 0–240 for female; 2nd 237–381 for male, 241–390 for female; 3rd 382–1200 for male, 391–1311 for female. ^‖^ Energy intake quintile ranges (kcal): 1st 688–1745 for male, 593–1613 for female; 2nd 1746–2097 for male, 1614–1942 for female; 3rd 2098–2467 for male, 1943–2285 for female; 4th 2468–3013 for male, 2286–2692 for female; 5th 3014–6413 for male, 2693–6495 for female. ^¶^ AHEI quintile ranges (points): 1st 7–16 for male, 7–16 for female; 2nd 17–19 for male, 17–19 for female; 3rd 20–22 for male, 20–22 for female; 4th 23–25 for male, 23–25 for female; 5th 26–34 for male, 26–35 for female. ** Daily consumption of juices, lemonades, hot chocolate, toffee, licorice, dried fruit (e.g., raisins), sweets, hard pastilles, or candy without xylitol, chocolate, or filled biscuits. ^††^ Systolic blood pressure ≥130 mmHg, or diastolic blood pressure ≥85 mmHg, or use of antihypertensive medication. ^‡‡^ SOC quartile ranges (score):1st 1.50–5.00 for male, 2.25–4.83 for female; 2nd 5.01–5.50 for male, 4.84–5.50 for female; 3rd 5.51–6.00 for male, 5.51–6.00 for female; 4th 6.01–7.00 for male, 6.01–7.00 for female.

**Table 3:** Self-report IWL during the previous year in men and women by potential determinants (*n* = 4525).

	Men and Women (*n* = 4525)					Men (*n* = 2147)					Women (*n* = 2378)					*p* for Inter-Action by Sex
	Age and Sex-Adjusted		Full Model *			Age-Adjusted		Full Model *			Age-Adjusted		Full Model *			
Determinants	*n*	(%)	*n*	OR	95% CI	*n*	(%)	*n*	OR	95% CI	*n*	(%)	*n*	OR	95% CI	
**Socio-demographic factors**																
Age (years)	4525		3749			2147		1780			2378		1969			**0.05**
30–39	1227	14.8	1055	1		583	9.43	489	1		644	19.6	566	1		
40–49	1327	13.7	1119	**0.75**	**0.58–0.97**	629	11.9	535	1.05	0.69–1.60	698	15.3	584	**0.58**	**0.42–0.82**	
50–59	1176	13.1	970	**0.62**	**0.46–0.83**	574	10.3	475	0.68	0.42–1.09	602	15.6	495	**0.56**	**0.38–0.82**	
60–69	795	8.70	605	**0.42**	**0.29–0.62**	361	9.42	281	0.64	0.35–1.15	434	8.29	324	**0.32**	**0.19–0.53**	
*p* for trend ^†^		**0.0002**					0.83					**<0.0001**				
Education (%)	4513		3749			2141		1780			2372		1969			0.23
Low	1450	11.3	1108	1		691	9.67	530	1		759	13.0	578	1		
Intermediate	1603	12.6	1366	1.14	0.87–1.50	881	9.43	753	1.03	0.68–1.56	722	15.8	613	1.27	0.89–1.81	
High	1460	15.0	1275	**1.40**	**1.07–1.85**	569	12.7	497	1.53	0.97–2.40	891	16.8	778	1.35	0.95–1.92	
*p* for heterogeneity		**0.02**					0.11					0.13				
**Lifestyle related factors**																
BMI (kg/m^2^)	4525		3749			2147		1780			2378		1969			**0.01**
<25	1746	7.37	1455	1		692	3.83	569	1		1054	10.2	886	1		
25–29.9	1805	14.3	1517	1.30	0.95–1.78	1010	11.9	854	**3.07**	**1.71–5.50**	795	16.4	663	0.86	0.58–1.27	
≥30	974	20.4	777	1.49	0.97–2.30	445	17.1	357	**4.44**	**2.03–9.67**	529	23.7	420	0.87	0.51–1.49	
*p* for trend ^†^		**<0.0001**					**<0.0001**					**<0.0001**				
FFMI quintiles (fat free mass kg/m^2^) ^‡^	4382		3749			2095		1780			2287		1969			0.53
1st (lowest)	875	5.68	752	1		419	3.80	344	1		456	6.99	408	1		
2nd	877	9.62	745	1.45	0.98–2.15	419	7.63	357	1.35	0.68–2.70	458	11.2	388	1.59	0.98–2.57	
3rd	876	13.0	754	**1.62**	**1.10–2.40**	419	10.3	369	1.58	0.80–3.13	457	15.5	385	**1.77**	**1.09–2.90**	
4th	876	16.4	753	**1.95**	**1.29–2.95**	419	14.4	361	2.00	0.99–4.05	457	18.4	392	**1.99**	**1.18–3.36**	
5th	878	20.0	745	1.59	0.96–2.64	419	15.7	349	1.13	0.48–2.66	459	24.4	396	**2.20**	**1.14–4.25**	
*p* for trend		**<0.0001**					**<0.0001**					**<0.0001**				
Leisure-time physical activity	4498		3749			2133		1780			2365		1969			0.84
Low	1108	11.2	900	1		543	9.18	439	1		565	13.1	461	1		
Moderate	2533	13.4	2097	**1.35**	**1.04–1.74**	1117	10.8	919	1.45	0.96–2.19	1416	15.8	1178	1.30	0.94–1.81	
Regular vigorous training	857	14.1	752	1.31	0.96–1.79	473	11.0	422	1.39	0.85–2.26	384	17.1	330	1.31	0.86–1.99	
*p* for heterogeneity		0.10					0.55					0.19				
Sitting time tertiles ^§^ (min/day)	4363		3749			2082		1780			2281		1969			0.48
1st (lowest)	1444	11.2	1211	1		677	8.71	557	1		767	13.3	654	1		
2nd	1418	12.8	1225	1.18	0.92–1.52	693	10.3	604	1.40	0.93–2.12	725	15.2	621	1.10	0.79–1.52	
3rd	1501	15.2	1313	**1.38**	**1.08–1.77**	712	11.9	619	**1.58**	**1.04–2.40**	789	18.2	694	1.30	0.96–1.78	
*p* for trend		**0.001**					**0.05**					**0.008**				
Smoking	4510		3749			2140		1780			2370		1969			0.64
Never	2207	11.5	1860	1		779	8.16	663	1		1428	14.6	1197	1		
Former smoker	943	17.1	788	**1.45**	**1.13–1.88**	601	13.7	501	1.38	0.91–2.09	342	19.4	287	**1.48**	**1.05–2.09**	
Current smoker	1360	12.5	1101	1.25	0.98–1.59	760	9.87	616	1.46	0.98–2.17	600	14.7	485	1.10	0.80–1.50	
*p* for heterogeneity		**0.0001**					**0.004**					0.08				
Energy intake quintiles ^‖^ (kcal/day)	4221		3749			1975		1780			2246		1969			0.56
1st (lowest)	844	15.6	735	1		395	12.7	351	1		449	17.9	384	1		
2nd	844	13.2	755	0.81	0.60–1.10	395	11.7	358	1.10	0.68–1.78	449	14.4	397	**0.65**	**0.44–0.97**	
3rd	844	12.4	743	0.78	0.57–1.06	395	8.63	349	0.80	0.47–1.34	449	15.6	394	0.76	0.52–1.13	
4th	844	13.5	758	0.87	0.64–1.18	395	10.8	357	0.96	0.58–1.59	449	16.0	401	0.77	0.52–1.14	
5th	845	10.6	758	**0.66**	**0.48–0.91**	395	9.04	365	0.88	0.52–1.47	450	12.3	393	**0.55**	**0.36–0.84**	
*p* for trend		**0.008**					0.10					0.07				
AHEI quintiles ^¶^	4221		3749			1975		1780			2246		1969			0.70
1st (lowest)	759	8.53	653	1		373	6.85	323	1		386	9.95	330	1		
2nd	835	10.6	742	1.35	0.94–1.94	390	7.61	354	1.11	0.61–2.02	445	13.2	388	1.50	0.95–2.37	
3rd	970	12.4	870	1.36	0.96–1.92	436	10.6	393	1.37	0.78–2.41	534	14.1	477	1.35	0.87–2.10	
4th	787	14.1	704	**1.59**	**1.11–2.27**	369	12.0	338	1.65	0.93–2.92	418	15.9	366	1.48	0.93–2.37	
5th	870	19.1	780	**2.21**	**1.56–3.12**	407	15.6	372	**1.87**	**1.07–3.28**	463	22.2	408	**2.42**	**1.54–3.79**	
*p* for trend		**<0.0001**					**<0.0001**					**<0.0001**				
Daily consuming certain sugary products **	4513		3749			2143		1780			2370		1969			1.00
No	3499	14.1	2925	1		1571	11.5	1310	1		1928	16.4	1615	1		
Yes	1014	8.89	824	**0.73**	**0.56–0.96**	572	7.12	470	0.76	0.51–1.13	442	10.5	354	0.73	0.50–1.07	
*p* for heterogeneity		**<0.0001**					**0.003**					**0.002**				
Sleep duration (hours)	4224		3749			1981		1780			2243		1969			0.03
≤6	583	13.3	491	1		316	13.9	266	1		267	12.2	225	1		
7–8	3170	13.3	2866	0.93	0.69–1.25	1509	10.1	1381	**0.63**	**0.42–0.95**	1661	16.0	1485	1.33	0.85–2.06	
≥9	471	11.6	392	0.94	0.63–1.41	156	9.63	133	0.79	0.40–1.57	315	13.1	259	1.22	0.71–2.11	
*p* for heterogeneity		0.60					0.13					0.14				
**Somatic health**																
Fs-triglycerides (mmol/L)	4510		3749			2142		1780			2368		1969			0.83
<1.7	3095	12.2	2584	1		1267	9.70	1044	1		1828	14.4	1540	1		
≥1.7	1415	14.5	1165	0.85	0.67–1.07	875	11.3	736	0.81	0.57–1.15	540	18.2	429	0.95	0.68–1.32	
*p* for heterogeneity		**0.04**					0.23					**0.04**				
Fs-HDL (mmol/L)	4510		3749			2142		1780			2368		1969			0.67
≥1.03 in men or ≥1.29 in women	2968	11.2	2478	1		1477	9.41	1235	1		1491	12.8	1243	1		
<1.03 in men or <1.29 in women	1542	16.2	1271	**1.33**	**1.07–1.65**	665	12.5	545	1.17	0.81–1.68	877	19.4	726	**1.43**	**1.08–1.88**	
*p* for heterogeneity		**<0.0001**					**0.03**					**<0.0001**				
Elevated blood pressure ^††^	4525		3749			2147		1780			2378		1969			0.77
No	1995	11.8	1691	1		764	10.0	642	1		1231	13.2	1049	1		
Yes	2530	13.9	2058	1.02	0.81–1.27	1383	10.6	1138	0.86	0.60–1.23	1147	17.5	920	1.17	0.88–1.57	
*p* for heterogeneity		**0.05**					0.68					**0.008**		0.28		
T2D	4525		3749			2147		1780			2378		1969			0.11
No	4365	12.6	3633	1		2064	9.87	1715	1		2301	15.0	1918	1		
Yes	160	23.0	116	1.60	0.99–2.58	83	23.2	65	**2.16**	**1.13–4.15**	77	22.2	51	1.07	0.52–2.21	
*p* for heterogeneity		0.0001					**0.0001**					0.09				
Knee or hip osteoarthritis	4467		3749			2131		1780			2336		1969			**0.03**
No	4249	12.6	3582	1		2020	9.71	1702	1		2229	15.3	1880	1		
Yes	218	20.3	167	1.53	0.99–2.38	111	21.5	78	**2.27**	**1.21–4.29**	107	18.6	89	1.04	0.55–1.96	
*p* for heterogeneity		**0.001**					**0.0001**					0.36				
**Psychological factors**																
SOC quartiles ^‡‡^	4314		3749			2023		1780			2291		1969			0.42
1st (highest)	1133	12.9	1003	1		543	11.2	500	1		590	14.3	503	1		
2nd	1127	13.7	1007	1.00	0.76–1.30	558	11.3	502	1.03	0.68–1.56	569	16.0	505	1.00	0.70–1.43	
3rd	1045	12.3	923	0.83	0.62–1.10	428	9.13	377	**0.59**	**0.35–0.97**	617	14.9	546	0.97	0.68–1.38	
4th (lowest)	1009	13.5	816	1.06	0.79–1.42	494	10.6	401	0.99	0.63–1.58	515	16.1	415	1.09	0.74–1.61	
*p* for trend		0.95					0.51					0.52				
Concerns about one’s appearance	4471		3749			2127		1780			2344		1969			0.07
Does not feel that looks any worse than used to	3729	12.6	3144	1		1891	9.93	1603	1		1838	15.1	1541	1		
Concerns about one’s appearance	742	15.4	605	1.10	0.84–1.44	236	14.5	177	1.53	0.93–2.53	506	16.8	428	0.97	0.70–1.34	
*p* for heterogeneity		**0.04**					**0.03**					0.35				
Concerns about one’s health	4480		3749			2125		1780			2355		1969			0.93
Not worried about their health more than usually	3097	12.3	2617	1		1527	10.1	1296	1		1570	14.3	1321	1		
Concerns about one’s health	1383	14.5	1132	1.00	0.80–1.25	598	11.3	484	0.91	0.62–1.34	785	17.3	648	1.10	0.83–1.46	
*p* for heterogeneity		**0.05**					0.42					0.06				

IWL, Intentional weight loss; *n*, Number of subjects in respective category; OR, Odds ratio; CI, Confidence interval; BMI, Body mass index; FFMI, Fat free mass index; AHEI, Alternate Healthy Eating Index; Fs-, Fasting serum; HDL, High density lipoprotein; T2D, Type 2 diabetes; SOC, Sense of coherence. Bolded results are statistically significant. * Adjusted for all the other variables in the table: sex (only when men and women analyzed together), age (continuous), education, BMI (continuous), FFMI (continuous as quintiles), leisure-time physical activity, sitting time (continuous as tertiles), smoking, energy intake (continuous as quintiles), AHEI (continuous as quintiles), daily consuming certain sugary products, sleep duration, fs-triglycerides, fs-HDL, elevated blood pressure, T2D, osteoarthritis, SOC (continuous as quartiles), concerns about one’s appearance, and concerns about one’s health. ^†^ Trend for continuous variable. ^‡^ FFMI quintile ranges (fat free mass kg/m^2^): 1st 14.3–19.5 for male, 11.1–16.3 for female; 2nd 19.6–20.5 for male, 16.4–17.2 for female; 3rd 20.6–21.4 for male, 17.3–18.1 for female; 4th 21.5–22.6 for male, 18.2–19.2 for female; 5th 22.7–29.4 for male, 19.3–24.2 for female. ^§^ Sitting time tertile ranges (min): 1st 0–236 for male, 0–240 for female; 2nd 237–381 for male, 241–390 for female; 3rd 382–1200 for male, 391–1311 for female. ^‖^ Energy intake quintile ranges (kcal): 1st 688–1745 for male, 593–1613 for female; 2nd 1746–2097 for male, 1614–1942 for female; 3rd 2098–2467 for male, 1943–2285 for female; 4th 2468–3013 for male, 2286–2692 for female; 5th 3014–6413 for male, 2693–6495 for female. ^¶^ AHEI quintile ranges (points): 1st 7–16 for male, 7–16 for female; 2nd 17–19 for male, 17–19 for female; 3rd 20–22 for male, 20–22 for female; 4th 23–25 for male, 23–25 for female; 5th 26–34 for male, 26–35 for female. ** Daily consuming juices, lemonades, hot chocolate, toffee, licorice, dried fruit, e.g., raisins, sweets, hard pastilles, or candy without xylitol, chocolate, or filled biscuits. ^††^ Systolic blood pressure ≥130 mmHg, or diastolic blood pressure ≥85 mmHg, or use of antihypertensive medication. ^‡‡^ SOC quartile ranges (score):1st 1.50–5.00 for male, 2.25–4.83 for female; 2nd 5.01–5.50 for male, 4.84–5.50 for female; 3rd 5.51–6.00 for male, 5.51–6.00 for female; 4th 6.01–7.00 for male, 6.01–7.00 for female.

## Supplementary Materials

The following is available online at https://www.mdpi.com/2072-6643/11/8/1789/s1, Table S1: Self-report dieting attempts and IWL during the previous year by interaction of BMI and selected determinants.
