# Supplementary material for: Self-Report Dieters: Who Are They?
Source: Nutrients. 2019 Aug 2;11(8):1789. doi: 10.3390/nu11081789 (PMC6723801; doi:10.3390/nu11081789)
Supplement: Supplementary file 1 [file nutrients-11-01789-s001.pdf]

Supplementary Table S1. Self-report dieting attempts and IWL during the previous year by interaction of BMI and selected determinants (n=3749)*.

	**Dieting attempts**						**IWL**					
	**BMI<25 (*n*=1455)**		**BMI=25-29.9 (*n*=1517)**		**BMI≥30 (*n*=777)**		**BMI<25 (*n*=1455)**		**BMI=25-29.9 (*n*=1517)**		**BMI≥30 (*n*=777)**	
**Determinants**	**OR**	**95% CI**	**OR**	**95% CI**	**OR**	**95% CI**	**OR**	**95% CI**	**OR**	**95% CI**	**OR**	**95% CI**
Sex												
Men	1		1		1		1		1		1	
Women	**4.11**	**2.84-5.95**	**2.47**	**1.94-3.13**	**1.49**	**1.09-2.02**	**2.92**	**1.80-4.74**	**1.38**	**1.00-1.89**	1.31	0.89-1.91
p for interaction						**<.0001**						**0.01**
Age (years)												
30-39	1		1		1		1		1		1	
40-49	**0.71**	**0.51-0.97**	1.00	0.74-1.35	0.92	0.57-1.49	**0.67**	**0.44-1.03**	0.74	0.50-1.08	0.99	0.57-1.73
50-59	**0.42**	**0.28-0.64**	**0.63**	**0.45-0.86**	0.76	0.48-1.20	**0.51**	**0.29-0.88**	**0.58**	**0.38-0.88**	0.90	0.53-1.53
60-69	**0.26**	**0.14-0.50**	**0.56**	**0.38-0.81**	**0.55**	**0.33-0.92**	**0.43**	**0.19-0.95**	**0.39**	**0.23-0.67**	0.59	0.31-1.11
p for interaction						0.23						0.82
Education												
Low	1		1		1		1		1		1	
Intermediate	1.17	0.77-1.79	0.93	0.70-1.24	1.24	0.87-1.77	1.02	0.58-1.79	0.94	0.64-1.40	1.52	0.98-2.35
High	**1.51**	**1.01-2.26**	1.33	0.99-1.79	1.16	0.78-1.70	1.39	0.82-2.36	1.29	0.87-1.92	1.37	0.86-2.18
p for interaction						0.43						0.46
Leisure-time physical activity												
Low	1		1		1		1		1		1	
Moderate	1.13	0.78-1.64	**1.34**	**1.01-1.79**	1.38	0.98-1.92	1.33	0.80-2.21	1.27	0.86-1.88	1.23	0.82-1.86
Regular vigorous training	1.23	0.80-1.90	**1.57**	**1.11-2.22**	**1.89**	**1.15-3.09**	1.14	0.62-2.06	1.27	0.79-2.02	1.28	0.72-2.28
p for interaction						0.79						0.98
Smoking												
Never	1		1		1		1		1		1	
Former smoker	1.24	0.83-1.84	1.06	0.80-1.41	**1.85**	**1.27-2.70**	1.21	0.71-2.07	**1.60**	**1.09-2.34**	1.48	0.96-2.28
Current smoker	1.01	0.73-1.39	0.76	0.58-1.00	0.88	0.60-1.28	1.33	0.88-2.01	1.28	0.89-1.84	1.03	0.65-1.63
p for interaction						0.12						0.77
Energy intake quintiles † (kcal/day)												
1st (lowest)	1		1		1		1		1		1	
2nd	0.67	0.44-1.02	1.10	0.78-1.55	0.79	0.49-1.26	**0.48**	**0.27-0.86**	0.92	0.59-1.45	1.13	0.63-2.01
3rd	**0.57**	**0.37-0.88**	0.78	0.55-1.12	0.88	0.55-1.40	**0.51**	**0.29-0.91**	0.86	0.54-1.38	1.02	0.57-1.82
4th	0.67	0.44-1.02	0.63	0.44-0.90	1.20	0.74-1.94	0.71	0.42-1.21	0.64	0.40-1.05	1.52	0.86-2.67
5th	**0.43**	**0.26-0.69**	**0.56**	**0.39-0.79**	0.99	0.61-1.59	**0.53**	**0.29-0.97**	0.71	0.44-1.12	0.75	0.41-1.37
P for interaction						**0.02**						0.15
AHEI quintiles ‡												

1st (lowest)	1		1		1		1		1		1	
2nd	1.33	0.84-2.09	1.43	0.97-2.11	1.35	0.79-2.29	1.48	0.78-2.81	1.20	0.69-2.07	1.59	0.79-3.19
3rd	1.22	0.78-1.93	**1.65**	**1.13-2.40**	1.51	0.92-2.48	1.74	0.94-3.23	1.38	0.82-2.32	1.04	0.53-2.06
4th	1.37	0.85-2.20	**1.71**	**1.16-2.52**	**2.29**	**1.36-3.85**	1.66	0.86-3.21	1.34	0.78-2.31	**2.01**	**1.03-3.93**
5th	**1.66**	**1.05-2.64**	**2.37**	**1.61-3.47**	**3.59**	**2.15-6.00**	**2.16**	**1.14-4.09**	**2.01**	**1.20-3.37**	**2.76**	**1.46-5.22**
p for interaction						0.31						0.61
Daily consuming certain sugary products [§]												
No	1		1		1		1		1		1	
Yes	0.76	0.53-1.09	**0.53**	**0.40-0.72**	1.29	0.86-1.93	0.62	0.38-1.03	**0.66**	**0.44-0.99**	1.03	0.62-1.71
p for interaction						**0.002**						0.31
Knee or hip osteoarthritis												
No	1		1		1		1		1		1	
Yes	0.31	0.04-2.37	1.65	0.99-2.77	0.70	0.42-1.17	-	-	**2.74**	**1.51-4.97**	1.12	0.59-2.12
p for interaction						**0.03**						**0.01**
SOC quartiles [||]												
1st (highest)	1		1		1		1		1		1	
2nd	0.84	0.57-1.24	1.01	0.74-1.37	1.15	0.74-1.78	0.78	0.47-1.29	1.25	0.83-1.86	0.88	0.53-1.45
3rd	1.03	0.70-1.50	1.18	0.86-1.61	1.09	0.70-1.70	0.81	0.48-1.35	0.98	0.64-1.52	0.66	0.39-1.13
4th (lowest)	1.40	0.93-2.09	**1.49**	**1.08-2.07**	1.52	0.98-2.37	1.30	0.77-2.17	1.23	0.79-1.92	0.73	0.43-1.24
p for interaction						0.97						0.22
Concerns about one's appearance												
Does not feel that looks any worse than used to	1		1		1		1		1		1	
Concerns about one's appearance	**1.64**	**1.14-2.35**	**1.41**	**1.04-1.93**	0.90	0.62-1.31	**1.71**	**1.07-2.72**	1.23	0.82-1.85	0.70	0.44-1.11
p for interaction						0.06						**0.02**
Concerns about one's health												
Not worried about their health more than usually	1		1		1		1		1		1	
Concerns about one's health	1.05	0.76-1.45	**1.27**	**1.00-1.61**	1.24	0.90-1.70	0.83	0.53-1.31	1.05	0.75-1.45	1.12	0.77-1.63
p for interaction						0.62						0.58

IWL, Intentional weight loss; BMI, Body mass index; *n*, Number of subjects in respective category; OR, Odds ratio; CI, Confidence interval; AHEI, Alternate Healthy Eating Index; SOC, Sense of coherence; FFMI, Fat free mass index; Fs-, Fasting serum; HDL, High density lipoprotein. Bolded results are statistically significant. * Adjusted for sex (only when men and women analyzed together), age (continuous), education, BMI (continuous), FFMI (continuous as quintiles), leisure-time physical activity, sitting time (continuous as tertiles), smoking, energy intake (continuous as quintiles), AHEI (continuous as quintiles),

daily consuming certain sugary products, sleep duration, fs-triglycerides, fs-HDL, elevated blood pressure, type 2 diabetes, osteoarthritis, SOC (continuous as quartiles), concerns about one's appearance, concerns about one's health. † Energy intake quintile ranges (kcal): 1st 688-1745 for male, 593-1613 for female; 2nd 1746-2097 for male, 1614-1942 for female ; 3rd 2098-2467 for male, 1943-2285for female; 4th 2468-3013 for male, 2286-2692 for female; 5th 3014-6413 for male, 2693-6495 for female. ‡ AHEI quintile ranges (points): 1st 7-16 for male, 7-16 for female; 2nd 17-19 for male, 17-19 for female ; 3rd 20-22 for male, 20-22 for female; 4th 23-25 for male, 23-25 for female; 5th 26-34 for male, 26-35 for female. § Daily consuming juices, lemonades, hot chocolate, toffee, liquorice, dried fruit, e.g. raisins, sweets, hard pastilles, or candy without xylitol, chocolate, or filled biscuits. || SOC quartile ranges (score):1st 1.50-5.00 for male, 2.25-4.83 for female; 2nd 5.01-5.50 for male, 4.84-5.50 for female; 3rd 5.51-6.00 for male, 5.51-6.00 for female; 4th 6.01-7.00 for male, 6.01-7.00 for female.
